# HDAC inhibitors as ferroptosis sensitizers in cancer: Epigenetic regulation of redox balance and iron metabolism

**DOI:** 10.17179/excli2026-9414

**Published:** 2026-07-08

**Authors:** Mubarak A. Alamri, Muhammad Afzal, Surya Nath Pandey, Obaid Afzal, Mohamed Ahmed Akela, A Rekha

**Affiliations:** 1 Department of Pharmaceutical Chemistry, College of Pharmacy, Prince Sattam bin Abdulaziz University, Al-Kharj 11942, Saudi Arabia; 2 Department of Pharmaceutical Sciences, Pharmacy Program, Batterjee Medical College, Jeddah 21442, Saudi Arabia; 3 Department of Pharmacology, Teerthanker Mahaveer College of Pharmacy, Teerthanker Mahaveer University, Moradabad 244001 Uttar Pradesh, India; 4 Department of Biology, College of Science and Humanities in Al-Kharj, Prince Sattam bin Abdulaziz University, Al-Kharj 11942, Saudi Arabia; 5 Dr DY Patil Medical College, Hospital and Research Centre, Pimpri, Pune 411018, India

**Keywords:** ferroptosis, histone deacetylase inhibitors, GPX4, SLC7A11, iron metabolism, epigenetic regulation

## Abstract

The evasion of programmed cell death significantly contributes to therapeutic failure in cancer, with resistance to apoptosis being the most prevalent form of resistance in multidrug-refractory diseases. Ferroptosis, an iron-dependent, non-apoptotic form of regulated cell death characterized by the lethal accumulation of lipid peroxides, represents a pharmacologically significant vulnerability in cancers that are resistant to apoptosis and tolerant to drugs. The resistance to ferroptosis, induced by the aberrant overexpression of the epigenetic enzyme histone deacetylases (HDACs) and the sustained transcriptional activity of key antiferroptotic targets, particularly GPX4 and SLC7A11, is enforced through epigenetic mechanisms. This review examines the extant preclinical and translational data, demonstrating that HDAC inhibitors predispose cancer cells to ferroptosis through four mechanistically convergent pathways. These pathways include the transcriptional silencing of SLC7A11 and subsequent glutathione depletion, disruption of intracellular iron homeostasis via ferroportin downregulation, enhancement of mitochondrial ROS-induced lipid peroxidation, and suppression of the HDAC3-NRF2-GPX4 antiferroptotic axis. The specific roles of HDAC1, HDAC3, and HDAC10 in colorectal, lung, gastric, and hematological cancers are elucidated. Additionally, the review discusses hybrid molecules of HDAC-ferroptosis, combination strategies with GPX4 inhibitors, and immunochemotherapy. Considerations such as isoform selectivity, biomarker development, and clinical translation are addressed, highlighting HDAC inhibitor-mediated ferroptosis sensitization as a promising strategy to overcome drug resistance in cancer.

See also the graphical abstract(Fig. 1).

## Introduction

One of the most challenging issues in biomedical research globally is the therapeutic failure of cancer. This complexity is heightened by mechanisms such as the evasion of programmed cell death (PCD) by malignant cells, which occurs due to the adaptive reorganization of survival pathways, making the disease system even more difficult to manage (Bian et al., 2025[2]; Sato et al., 2024[39]). Traditional anticancer methods of chemotherapy, targeted therapy, and radiotherapy are largely based on the induction of apoptosis (Dai et al., 2024[5]; Singh et al., 2025[44]). Nevertheless, there has been an increasing need to identify alternative regulated cell death pathways that are mechanistically independent of apoptosis and retain cytotoxic efficacy in tumors that are resistant to apoptosis through mutation in TP53, excessive expression of anti-apoptotic members of the BCL-2 family, and caspase cascade dysregulation (Darbandi et al., 2026[6]; Quaranto et al., 2025[37]).

Ferroptosis is an iron-dependent, non-apoptotic type of regulated cell death initially described in 2012 is executed by the fatal accumulation of reactive oxygen species (ROS)-driven lipid peroxides at the stage of a polyunsaturated fatty acid (PUFA)-containing phospholipid in cellular membranes (Zhou et al., 2024[66]; Zille et al., 2019[69]). The main molecular defense system against ferroptosis is the glutathione peroxidase 4 (GPX4)-glutathione (GSH)-SLC7A11 axis where system Xc^-^, cystine/glutamate antiporter, imports cystine extracellularly to be converted into intracellular GSH, and GPX4 uses GSH as an antioxidant to reduce lethal lipid hydroperoxide to non-toxic lipid alcohols (Lin et al., 2020[29]). Most importantly, SLC7A11 is upregulated in various cancers, in which it imparts ferroptotic resistance and contributes to multidrug resistance through preserving GSH-dependent GPX4 activity (Chen et al., 2026[3]). Mesenchymal and drug-tolerant persister cancer cells, which are pivotal in therapeutic relapse, exhibit a significant dependence on the GPX4-SLC7A11 axis for survival. Consequently, ferroptosis presents a potential vulnerability in treatment-resistant tumor populations (Zhang et al., 2024[59]).

Histone deacetylases (HDACs) are epigenetic enzymes which remove acetyl groups at lysine residues on histone tails, which facilitate condensing chromatin and silencing transcription of cell cycle regulatory genes, oxidative stress response genes, and genes regulating cell death (Hassell, 2019[11]; Zhou et al., 2025[67]). The overexpression and aberrant activity of histone deacetylases (HDACs), particularly class I isoforms such as HDAC1, HDAC2, HDAC3, and HDAC10, have been implicated in the epigenetic regulation of ferroptosis resistance. This is achieved by maintaining the transcriptional activity of antiferroptotic targets, including SLC7A11 and GPX4, in both hematological and solid malignancies (Jin et al., 2025[21]). HDAC10 inhibits miR- 223-5p expression in non- small cell lung cancer, which suppresses SLC7A11, thus preserving ferroptosis resistance in a three-node epigenetic-post-transcriptional circuit (Shi et al., 2024[42]). Recent studies have indicated that panobinostat and vorinostat, both pan-HDAC inhibitors, exhibit a cooperative effect with GPX4 inhibitors. This collaboration selectively induces the death of drug-tolerant pan-persister cells, while sparing parental non-persister cells. The mechanism involves the sensitization of mitochondrial ROS-mediated ferroptosis, which specifically targets the persister cells (Higuchi et al., 2026[15]).

Recent data on HDAC inhibitors in cancer models, both in vitro and in vivo, suggest that four distinct pathways contribute to the sensitization of cancer cells to ferroptosis (Zhang et al., 2021[60]). These pathways include the transcriptional repression of SLC7A11 and subsequent depletion of GSH (Zhang et al., 2021[60]), disruption of intracellular iron homeostasis through the downregulation of ferroportin and expansion of labile iron pools (Oliveira et al., 2021[33]), mitochondrial overproduction of reactive oxygen species (ROS) leading to lipid peroxidation (Higuchi et al., 2026[15]), and the epigenetic silencing of the HDAC3-NRF2-GPX4 antiferroptotic axis (Jin et al., 2025[21]). Despite the burgeoning body of mechanistic evidence, a comprehensive narrative review that synthesizes the roles of isoforms, cancer type-specific preclinical evidence, and the translational and combination therapeutic potentials of HDAC inhibition as a ferroptotic sensitizing strategy has yet to be published. This review addresses this gap by summarizing existing mechanistic, pharmacological, and translational data, with a particular focus on the epigenetic rearrangement of redox balance and iron metabolism as the central axis of HDAC inhibitor-induced ferroptosis sensitization in cancer.

## Ferroptosis: Core Molecular Machinery

Ferroptosis is a mechanistically different form of apoptosis, necroptosis, and autophagy, which is characterized by the mandatory simultaneous occurrence of three biochemical hallmarks: the distribution of iron-dependent production of reactive oxygen species (ROS), the peroxidation of polyunsaturated fatty acid (PUFA)-containing phospholipids in cell membranes, and the inability or inhibition of lipid hydroperoxide repair mechanisms (Pla et al., 2024[35]; Wu et al., 2025[50]). Understanding these molecular pathways is essential for contextualizing how HDAC inhibitors epigenetically reconfigure each component of this system, thereby rendering cancer cells vulnerable to ferroptotic cell death.

### The GPX4-GSH-SLC7A11 axis

The primary and most extensively investigated mechanism of defense against ferroptosis is the SLC7A11-glutathione (GSH)-glutathione peroxidase 4 (GPX4) axis. SLC7A11 (synonymous with xCT) is the catalytic subunit of the heterodimeric system Xc- transporter, which replaces extracellular cystine with intracellular glutamate in a sodium independent, chloride-dependent process (Chen et al., 2026[3]). The imported cystine is then reduced quickly to cysteine in the cell interior that is the rate-limiting substrate in the manufacture of GSH in the cell through γ-glutamylcysteine synthetase and GSH synthase. GSH is then used as a reducing cofactor to catalyze the ability of GPX4, a phospholipid-specific glutathione peroxidase, to convert lipid hydroperoxides (Lipid-OOH) into non-toxic lipid alcohols (Lipid-OH), which are the actual agents of ferroptotic membrane damage directly neutralized by GSH (Ou et al., 2024[34]).

The gene SLC7A11 is overexpressed in various cancers, including colorectal, gastric, breast, and lung cancer. In these malignancies, elevated levels of cystine import are crucial for maintaining high levels of glutathione (GSH) and glutathione peroxidase 4 (GPX4) activity. This biochemical environment enables cancer cells to sustain a ferroptosis-resistant redox state, thereby conferring resistance to a broad spectrum of chemotherapeutic agents (Cui et al., 2026[4]). Intracellular GSH and GPX4 inactivation, and the resultant ferroptotic cell death in a variety of cancer models, is induced by genetic knockdown or pharmacological inhibition of SLC7A11 (Wang et al., 2024[48]). The oncogenic coactivator YAP and the transcription factor NRF2 both promote the transcription of SLC7A11 during conditions of oxidative and mechanical stress. This results in a series of oncogenic signals that converge on SLC7A11, thereby enhancing the resistance of tumor cells to ferroptosis (Wang et al., 2024[48]).

### Iron metabolism and the labile iron pool

Iron serves as the catalyst for ferroptosis. The labile iron pool (LIP) may contain free ferrous iron (Fe2+), which participates in Fenton-type reactions to produce highly reactive hydroxyl radicals. These radicals are generated through hydrogen peroxide and subsequently induce and amplify lipid peroxidation in phospholipids within the membrane (Jiang et al., 2025[20]; Wen et al., 2019[49]). The regulation of the labile iron pool (LIP) is governed by a dynamic network of transport, storage, and regulatory proteins. Cellular iron uptake is mediated through transferrin receptor 1 (TFRC), while ferritin, composed of the FTH1/FTL heteropolymeric cage, sequesters excess intracellular iron to mitigate the potential for Fenton chemistry. Ferroportin (SLC40A1) facilitates the export of iron from the cell, and its downregulation directly increases the size of the LIP, thereby heightening susceptibility to ferroptosis.

Ferritinophagy, the selective autophagic degradation of ferritin facilitated by the cargo receptor NCOA4, redistributes the iron sequestered in ferritin back into the cytosolic labile iron pool (LIP), thereby enhancing reactive oxygen species (ROS) generation and ferroptotic signaling (Jiang et al., 2025[20]). The cancer cells, especially drug-tolerant mesenchymal cells and persister populations are highly dependent on iron as indicated by an upregulated TFRC expression, low ferroportin activity, and an increased rate of ferritinophagic iron-dependent ferroptotic death (Wang et al., 2024[48]). This dependency on iron represents a selective vulnerability that can be therapeutically exploited by agents that destabilize iron homeostasis in conjunction with ferroptosis inducers.

### ACSL4, LPCAT3, and lipid peroxidation execution

The process of ferroptosis involves the utilization of a substrate, specifically polyunsaturated fatty acid (PUFA)-containing phospholipids, which are esterified at the sn2 position of the glycerol backbone. These phospholipids predominantly consist of phosphatidylethanolamines (PEs) that incorporate arachidonic acid (AA, C20:4) or adrenic acid (AdA, C22:4) (Jiang et al., 2025[20]). This ferroptotic substrate is formed in two consecutive steps by enzymes. To begin with, free AA and AdA are first activated to their coenzyme A thioesters (AA-CoA and AdA-CoA) by acyl-CoA synthetase long-chain family member 4 (ACSL4). Second, these activated PUFAs are then esterified by lysophosphatidylcholine acyltransferase 3 (LPCAT3) into membrane PE phospholipids to form the oxidizable substrate pool (AA-PE and AdA-PE) (Doll et al., 2017[8]). These PUFA-PEs are then oxidized by 15-lipoxygenase (15-LOX/ALOX15) to produce lipid hydroperoxides (PE-AA-OOH and PE-AdA-OOH) capable of carrying out membrane damage and cell death under the inhibition of GPX4 or the depletion of the GSH (Jia et al., 2023[19]).

The expression of ACSL4 serves as a robust positive predictor of sensitivity to ferroptosis. Specifically, cells lacking ACSL4 exhibit significant resistance to ferroptosis induced by GPX4 inhibitors, whereas the overexpression of ACSL4 increases cellular susceptibility to ferroptotic stimuli (Doll et al., 2017[8]). Elevated expression of ACSL4 has been documented in various human malignancies, and the production of lipid peroxidation substrates mediated by ACSL4 represents a targetable step in the ferroptosis induction pathway (Jiang et al., 2025[20]).

### Secondary ferroptosis defense systems: FSP1 and GCH1/BH4

In addition to the canonical GPX4-GSH axis, two alternative GPX4-independent mechanisms are employed to inhibit ferroptosis in cancer cells. The first of these is the ferroptosis suppressor protein 1 (FSP1)/coenzyme Q10 (CoQ10) axis. FSP1 functions as an NAD(P)H-dependent oxidoreductase, facilitating the reduction of oxidized CoQ10 to CoQH2 at the plasma membrane. This process generates a lipophilic radical-trapping antioxidant (RTA) that impedes the propagation of lipid peroxides without the involvement of GSH and GPX4 (Lee and Roh, 2023[25]). FSP1 increases ferroptosis resistance in cancer cells: its knockout increases phospholipid oxidation and makes cells susceptible to GSH depletion as well as to GPX4 inhibition, whereas its overexpression provides a strong resistance despite the pharmacological inhibition of GPX4 (Silver et al., 2026[43]).

The second system under consideration is the GCH1/tetrahydrobiopterin (BH4) axis. GTP cyclohydrolase 1 (GCH1) is an enzyme that facilitates the synthesis of BH4, a potent endogenous lipophilic antioxidant. BH4 functions by inhibiting membrane lipid peroxidation independently of glutathione (GSH) through the direct scavenging of lipid radicals and indirectly by sustaining CoQ10 production via the provision of necessary precursors (Ding et al., 2025[7]). GCH1 genetic ablation sensitizes cancer cells resistant to drugs to ferroptosis inducers, whereas GCH1 expression makes cancer cells resistant to RSL3, erastin, and direct deletion of GPX4 - making GCH1/BH4 an autonomous, therapeutically significant ferroptosis resistance node in cancer biology (Qi et al., 2022[36]).

The ferroptotic molecular architecture constitutes a complex network of interconnected systems, including the GPX4-GSH-SLC7A11 axis, the iron-LIP-Fenton system, the ACSL4/LPCAT3/15-LOX lipid peroxidation cascade, and the FSP1/CoQ10 and GCH1/BH4 secondary defense systems. The aberrant maintenance of these systems, facilitated by HDAC activity, leads to the formation of a multi-layered ferroptosis-resistant phenotype in cancer cells. This review primarily focuses on the pharmacological premise of degrading this phenotype through HDAC inhibition.

## HDAC Biology in Cancer

### Classification and isoforms

The human HDAC family has 18 enzymes that can be classified into four structural classes depending on sequence homology, catalytic mechanism and localization. Class I HDACs (HDAC1, 2, 3, 8) are zinc-dependent, ubiquitously expressed nuclear enzymes which, to a great extent, regulate chromatin compaction and transcriptional repression via multiprotein co-repressor complexes (NuRD, Sin3A, and CoREST) formation (Hai et al., 2021[10]). Members of Class IIa (HDAC4, 5, 7, 9) and Class IIb (HDAC6, 10) are zinc-dependent enzymes, which dynamically alter their localization between the nucleus and cytoplasm and regulate gene expression and cytoskeleton assembly by deacetylation of non-histone proteins, such as α-tubulin and HSP90. (Hassell, 2019[11]). The sirtuins (SIRT1-7) are the class III HDACs and use NAD+ as the cofactor instead of zinc and regulate metabolic adjustments, DNA damage response, and mitochondrial activity by deacetylation of transcription factors such as p53, HIF-1α and FOXO proteins. Class IV The lone enzyme, HDAC11, has structural properties similar to both class I and II, and is emerging in the regulation of immunological functions in the tumor microenvironment (Zhang et al., 2025[64]).

### HDAC dysregulation in oncogenesis

The overexpression and recruitment of histone deacetylases (HDACs) are recognized as characteristic features of malignancy across various cancer types. Class I HDACs, especially HDAC1, HDAC2, and HDAC3, are transcriptionally overexpressed in colorectal, lung, gastric, hepatocellular, and hematological cancers, and in these cancers the activity of class I HDACs results in silencing of tumor suppressor genes such as p21, p53, PTEN, and RB1, via deacetylation of the promoter histone H3 (Yang et al., 2014[54]). In addition to histones, oncogenic signaling is enhanced by HDAC-mediated deacetylation of non-histone substrates: HDAC1/2 catalytic inactivation of p53, c-MYC, and p300 boosts tumor biology applications of isoform-specific HDAC activity (Hess et al., 2022[14]). HDAC3 serves to regulate NF-kB-dependent transcription, inhibit T-cell infiltration via CD8+ cells by silencing CXCL10, and to sustain oncogenic transcription programs by Wnt/β-catenin pathway deacetylation in cancer stem cells (Zhang et al., 2025[64]).

HDAC10 is a G2/M cell cycle transporter that regulates the mitosis of cells by modulating the expression of cyclin A2 and inhibits cancer invasion by modulating the expression of MMP2/MMP9, and its deficiency in non-small cell lung cancer is linked to the susceptibility to ferroptosis via the miR-223-5p/SLC7A11 signaling pathway (Shi et al., 2024[42]). Together, HDACs can sustain the ferroptotic-resistant transcriptome of cancer cells by stabilizing the expression of antiferroptotic targets - including SLC7A11 and GPX4, most badly - by silencing their negative regulators with epigenetics (Jin et al., 2025[21]).

### Clinically approved HDAC inhibitors

Regulatory approval has been granted for five histone deacetylase (HDAC) inhibitors for the treatment of cancer. Initially, vorinostat and romidepsin received authorization for use in relapsed or refractory cutaneous T-cell lymphoma. Subsequently, belinostat and panobinostat were approved for peripheral T-cell lymphoma and multiple myeloma, respectively. Finally, chidamide was approved in China as a class I-selective agent for peripheral T-cell lymphoma (Liang et al., 2023[28]). Class I and II isoforms are inhibited by pan-HDACi including vorinostat and panobinostat using zinc-chelating hydroxamic acid pharmacophores and have the greatest scope of ferroptosis-sensitizing in preclinical cancer models (Higuchi et al., 2026[15]). Class I-selective benzamides, such as entinostat and HDAC3-selective RGFP966, retain ferroptosis-sensitising activity at potentially reduced off-target toxicity than pan-inhibitors, providing a more specific pharmacological approach to solid tumor indications (Jin et al., 2025[21]). The presence of licensed HDACi-based ferroptosis sensitizers, characterized by well-defined pharmacokinetics, safety profiles, and clinical histories, facilitates the immediate translation of HDACi-based ferroptosis sensitization as a combination therapy in cancer treatment (Tian et al., 2025[47]).

## HDAC Inhibitors as Ferroptosis Sensitizers in Cancer: Mechanistic Evidence

Histone deacetylase (HDAC) inhibitors enhance the susceptibility of cancer cells to ferroptosis through four mechanistically distinct yet converging pathways. These include the transcriptional silencing of SLC7A11, leading to downstream glutathione (GSH) depletion; disruption of intracellular iron homeostasis via the downregulation of ferroportin and expansion of the labile iron pool (LIP); increased mitochondrial reactive oxygen species (ROS) and amplification of lipid peroxidation; and suppression of antiferroptosis mediated by the HDAC3-NRF2-GPX4 axis (Xiao et al., 2025[52]). These mechanisms operate in a context-specific manner and exhibit redundancy across various cancer types, collectively dismantling the epigenetically encoded ferroptosis-resistant phenotype of cancer cells.

### Transcriptional suppression of SLC7A11 and GSH depletion

The most profoundly described process by which HDAC inhibitors sensitize cancer cells to ferroptosis is transcriptional downregulation of the SLC7A11 gene that causes a lack of the importation of cystine and the intracellular depletion of GSH, as well as functional inactivation of GPX4. Vorinostat and entinostat treatment in EGFR-TKI-resistant lung adenocarcinoma cells are able to cause a significant decrease in the levels of SLC7A11 mRNA and protein, depleting intracellular GSH and increasing ferroptosis with erastin at sub-cytotoxic HDACi levels - showing that HDAC inhibition lowers the ferroptosis threshold without stimulating endogenous cell death on its own (Zhang et al., 2021[60]). Mechanistic evidence of SLC7A11 repression by HDACi entails inactivation of transcription factors (NRNF2 and ATF4) using acetylation, which activate and maintain SLC7A11 transcription in oxidative and metabolic stress settings, which paradoxically destroys the defense that cancer cells depend on to survive high ROS environments (Lee and Roh, 2022[24]).

Notably, class I HDAC inhibitors not only enhance ferroptosis induced by erastin in neuroblastoma, hepatoma, and fibrosarcoma cell lines but also have a neuroprotective effect on normal neurons - a selectivity that is explained by the fact that different HDAC isoforms are expressed in malignant and non-malignant cell lines (Lin et al., 2020[29]). This selectivity was also supported in gastric cancer cells wherein vorinostat and entinostat induced lipid peroxidation and ferroptosis via SLC7A11 suppression and GSH loss, even in cisplatin-resistant cell lines in which the resistance to ferroptosis through SLC7A11 had been the dominant mechanism of chemoresistance (Jenke et al., 2024[18]). The administration of HDAC inhibitors in acute myeloid leukemia cells resulted in the depletion of GSH through the downregulation of SLC7A11 expression. Furthermore, HDAC inhibitors modulated iron regulatory proteins in both solid and hematological malignancies, indicating that the targeting of SLC7A11 by HDAC inhibitors occurs across these cancer types (Bian et al., 2025[2]).

### Disruption of iron homeostasis via ferroportin downregulation

Another mechanically distinct type of HDACi-induced ferroptosis sensitization is an intracellular iron homeostasis reprogramming, which is not dependent on the SLC7A11 or GSH condition. Oliveira et al. showed that treatment of cells with SW13 adrenocortical carcinoma with a class I HDACi, romidepsin (FK228), induced an epithelial-to-mesenchymal transition (EMT), which was accompanied by an increase in total intracellular iron content by 29.5 percent (Oliveira et al., 2021[33]). This iron buildup was determined through mechanistic study to be based on transcriptional suppression of ferroportin (SLC40A1) - the exclusive iron export pore- to prevent intracellular iron export and ultimately increase the amount of LIP to produce Fenton-type ROS (Oliveira et al., 2021[33]). The heightened level of the iron regulatory protein (IRP1 and IRP2) binding as evidence of cellular detection of the iron surplus was also seen simultaneously, proving that the cells experience a bona fide alteration of iron homeostasis during HDAC inhibitor-induced EMT. The induction of ferroptosis following erastin treatment was notably augmented in cells in the mesenchymal state through HDACi pretreatment. Furthermore, there exists a direct causal relationship between iron accumulation induced by HDACi pretreatment and increased vulnerability to ferroptosis (Oliveira et al., 2021[33]).

This iron-based mechanism is particularly relevant to cancers that acquire mesenchymal characteristics during treatment such as cancer stem cells, circulating tumor cells, and drug-tolerant persister populations because the acquisition of epithelial-mesenchymal transition (EMT) increases iron dependency and predisposes these cells to ferroptosis (Rodriguez et al., 2022[38]). The HDAC3-Hippo/YAP-hepcidin axis also supports the role of HDAC3 in systemic and cellular iron control: deletion of HDAC3 in hepatocytes in mouse models led to a decrease in hepcidin expression with the aid of nuclear translocation of YAP, which causes iron overload and iron-induced ferroptotic liver damage - an observation that supports the validity of HDAC3-iron homeostasis (Meng et al., 2023[32]). Although this process was described in non-cancer liver tissue, it presents valuable mechanistic precedence to the role of HDAC3 in the regulation of intracellular iron content and iron overload in the ferroptosis of cancer cells.

### Mitochondrial ROS elevation and lipid peroxidation amplification

The third, independently mechanistically operating division of HDACi-mediated ferroptosis sensitization is by the increase in mitochondrial reactive oxygen species, regardless of GSH status or labile iron status. Higuchi et al. established that clinically approved pan-HDACi panobinostat and vorinostat potently forced total cellular ROS and more specifically mitochondrial ROS in drug-tolerant lung cancer persister cells as assessed by an increase in MitoSOX and CellROX fluorescent signals (Higuchi et al., 2026[15]). This increase in ROS was accompanied by slight inhibition of oxidative phosphorylation and antagonistic increase in OXPHOS gene expression, which is compatible with uncoupling of the electron transport chain in the mitochondrion as the pathophysiology behind the production of ROS. Notably, the concurrent administration of HDACi with the mitochondria-targeted antioxidant mitoTEMPO entirely abrogated ferroptotic sensitization induced by HDACi. This finding identifies mitochondrial ROS as the principal sensitizing agent in this cellular model, rather than GSH depletion or iron overload (Higuchi et al., 2026[15]).

The mitochondrial reactive oxygen species (ROS) pathway converges with the inhibition of glutathione peroxidase 4 (GPX4) to generate an excessive accumulation of lipid peroxidation. This accumulation cannot be mitigated by pre-ferroptotic cells, which are already reliant on iron and exhibit a low capacity for oxidative phosphorylation, due to the absence of GPX4. Panobinostat and vorinostat were combined with GPX4 inhibitor RSL3 to selectively kill persister cells in lung and melanoma and breast cancer models, but parental drug-sensitive cells were mostly spared (Higuchi et al., 2026[15]). The HDACi-ROS mechanism is also underpinned by the fact that SAHA-resistant cancer cell lines do not accumulate ROS after HDACi treatment, and sensitive cell lines exhibit a strong increase in caspase-independent ROS after HDACi treatment, proving that ROS induction is an active determinant of HDACi anticancer effects in addition to ferroptosis sensitization (Karaj et al., 2022[22]). Mitochondrial ROS is acting independently to oxidize membrane PUFA-phospholipids by non-enzymatic Fenton reactions and through activation of ACSL4-dependent enzymatic pathways of peroxidation; this is why greater lipid peroxidation is observed when HDACi-induced ROS is combined with GPX4 inhibition (Liu et al., 2023[30]).

### The HDAC3-NRF2-GPX4 antiferroptotic axis

**Figure 2:**
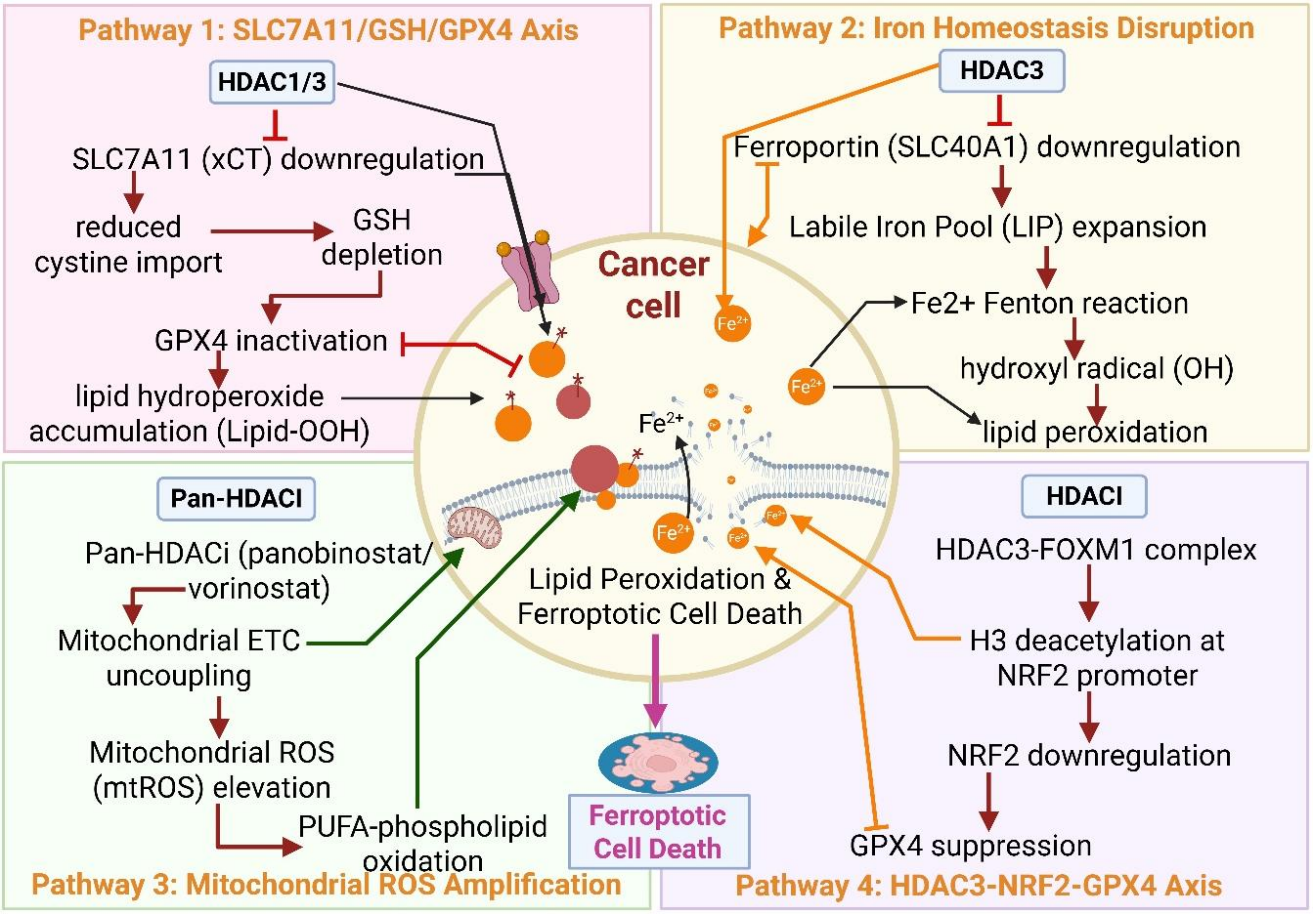
Epigenetic mechanisms by which HDAC inhibitors sensitize cancer cells to ferroptosis through four convergent molecular pathways. HDAC inhibition dismantles ferroptosis resistance via: (1) SLC7A11/GSH/GPX4 axis suppression, (2) ferroportin downregulation and labile iron pool expansion, (3) mitochondrial ROS amplification; and (4) the HDAC3-NRF2-GPX4 transcriptional cascade. All four pathways converge on lethal lipid peroxidation and ferroptotic cell death. Flat-headed arrows denote inhibition, pointed arrows denote activation. HDACi, histone deacetylase inhibitor; GSH, glutathione; GPX4, glutathione peroxidase 4; LIP, labile iron pool; mtROS, mitochondrial reactive oxygen species; NRF2, nuclear factor erythroid 2-related factor 2. Created with BioRender.com

The HDAC3-NRF2-GPX4 regulatory pathway is the most accurately defined sequence of epigenetic interactions in which the HDAC activity keeps ferroptosis resistance on the level of transcriptional gene control. Jin et al. found HDAC3 to be a central epigenetic inhibitor of ferroptosis in colorectal cancer by showing that selective HDAC3 inhibitor includes RGFP966, as well as post-genetic knockdown of HDAC3, were both able to significantly increase ferrous iron (Fe2+) levels in the cell, lipid ROS generation, and ferroptotic cell death (Jin et al., 2025[21]). Mechanistically, HDAC3 interacts with the NRF2 promoter together with the transcription factor FOXM1, where it catalyses histone H3 deacetylation at this locus, and constitutes NRF2 transcription in a permissive chromatin conformation. HDAC3 inhibition leads to the downregulation of NRF2 mRNA and protein that subsequently leads to a drop in the expression of GPX4- the major lipid hydroperoxide detoxification enzyme, effectively disabling the major defense mechanism of ferroptosis in CRC cells (Jin et al., 2025[21]). The mechanistic order of hierarchy rescue experiments, NRF2 knockdown ablated GPX4 downregulation after HDAC3 inhibition, GPX4 overexpression prevented cells in HDAC3 knockdown-ferroptosis, and ferrostatin-1 co-treatment showed ferroptotic as opposed to apoptotic cell death to be the working mechanism. The HDAC3-NRF2-GPX4 axis constitutes an epigenetic-transcriptional cascade in which HDAC3 activity serves as the upstream driver of ferroptosis resistance, NRF2 functions as the intermediate transcriptional relay, and GPX4 acts as the downstream effector. Each of these nodes is a target for pharmacological interventions against ferroptosis (Jin et al., 2025[21]). Additionally, another mechanism of NRF2 epigenetic regulation involves the acetylation and deacetylation of NRF2 itself, which modulates the activity of downstream antioxidant target genes such as SLC7A11, FTH1, and HMOX1, indicating that the action of HDAC3 extends beyond GPX4 alone (Yang et al., 2025[56]) (Figure 2(Fig. 2)).

## Isoform-Specific HDAC Roles in Ferroptosis Regulation

While the class of HDAC inhibitors possesses the capacity to sensitize cancer cells to ferroptosis, recent investigations have delineated the roles of specific HDAC isoforms in relation to distinct molecular nodes of ferroptosis resistance. Isogenic, precise, and detailed genetic and pharmacological studies have identified three isoforms: HDAC1, HDAC3, and HDAC10.

### HDAC1 and the FTH1-iron axis

HDAC1 controls ferroptosis sensitivity in colorectal cancer by transcriptionally regulating the major intracellular iron storage protein ferritin heavy chain 1 (FTH1). Huang et al. showed that ferroptosis vulnerability in CRC is controlled by the VCPKMT-VCP-HDAC1 regulatory axis: VCPKMT-mediated methylation of the AAA-ATPase VCP promotes its nuclear translocation in which VCP increases proteasomal degradation of HDAC1, which results in transcriptional repression of FTH1 (Huang et al., 2025[17]). Lowering FTH1 decreases the iron storage capacity of CRC cells which widen the labile iron pool (LIP) and increases cells susceptible to erastin-induced ferroptosis as shown by increased lipid peroxidation, heightened intracellular Fe2+, and typical morphological changes in the mitochondrion on transmission electron microscopy (Huang et al., 2025[17]). Rescue studies revealed that VCPKMT-induced ferroptosis susceptibility was reversed by FTH1 re-expression, and HDAC1 re-expression restored FTH1 levels and recovered ferroptosis resistance - HDAC1-FTH1 was a mechanistically validated epigenetic axis in iron-dependent ferroptosis resistance in CRC. A complementary study also found that lactylation at HDAC1 at the lysine 412 position in CRC cells stabilizes HDAC1 protein, which subsequently stabilizes FSP1 expression via the m6A-mediated mRNA stabilizing mechanism; HDACi treatment reverses HDAC1 lactylation, decreases FSP1 levels and increases CRC cell susceptibility to ferroptotic inducers such as RSL3, erastin and FINO2 (Yang et al., 2025[57]).

### HDAC3 and the NRF2-GPX4 transcriptional hierarchy

The isoform most comprehensively characterized in the regulation of cancer ferroptosis is HDAC3. Jin et al. (2025[21]) elucidated that HDAC3 epigenetically modulates the transcriptional level of NRF2 through promoter histone H3 deacetylation in colorectal cancer cells. This process enables NRF2 to maintain GPX4 expression levels, thereby establishing the linear HDAC3-NRF2-GPX4 ferroptosis resistance cascade (Jin et al., 2025[21]). RGFP966, a selective inhibitor of HDAC3, gave the strongest ferroptotic phenotype of any of the class I isoform inhibitors tested - by far - significantly stronger than HDAC1/2-selective inhibition - and was fully suppressed by KD of NRF2, or by GPX4 overexpression, demonstrating the mechanistic specificity of the HDAC3-NRF2-GPX4 axis (Jin et al., 2025[21]). It is interesting to note that selective inhibition of HDAC6 with tubastatin A did not result in any ferroptotic phenotype in these cells, which demonstrates the isoform specificity of ferroptosis regulation and may lead to ferroptosis sensitization with lower toxicity with HDAC3-targeted inhibitors than pan-HDACi (Jin et al., 2025[21]).

### HDAC10 and the H3K9ac-miR-223-5p-SLC7A11 circuit

**Figure 3:**
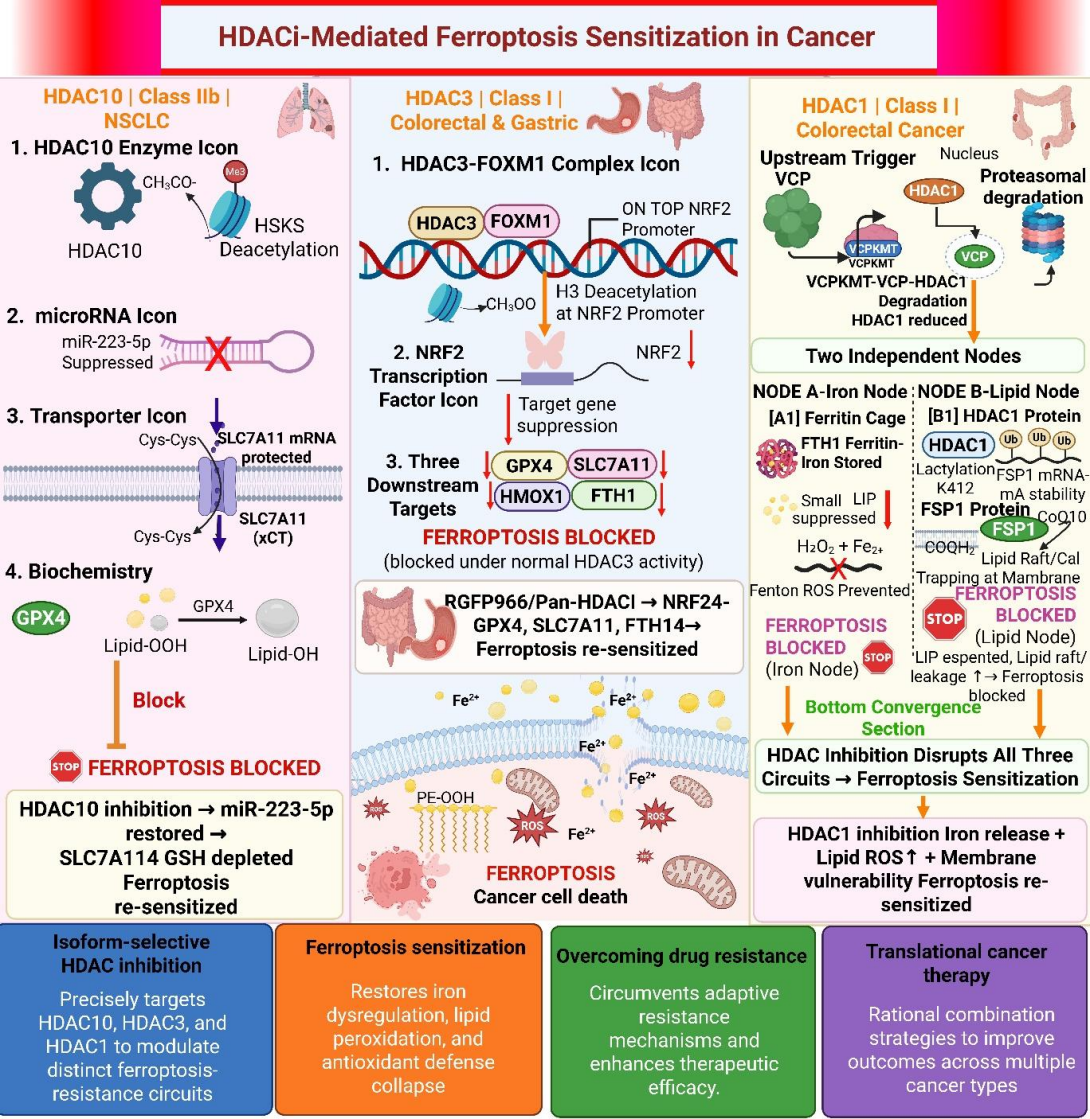
Isoform-specific HDAC regulatory circuits maintaining ferroptosis resistance across cancer types. HDAC10 sustains ferroptosis resistance in non-small cell lung cancer by deacetylating H3K9 at the miR-223-5p gene promoter, thereby suppressing miR-223-5p transcription and preserving SLC7A11 (xCT) expression, which maintains intracellular GSH levels and GPX4 activity to neutralize lipid hydroperoxides. In colorectal and gastric cancers, the HDAC3-FOXM1 complex deacetylates histone H3 at the NRF2 promoter to sustain NRF2-driven transcription of GPX4, SLC7A11, FTH1, and HMOX1, collectively establishing a multi-target antiferroptotic transcriptional program. In colorectal cancer, HDAC1 maintains ferroptosis resistance through two independent nodes FTH1-mediated iron sequestration that suppresses the labile iron pool, and lactylation at lysine-412 that stabilizes FSP1 mRNA via m6A modification, sustaining GPX4-independent lipid radical trapping via the FSP1-CoQ10 axis at the plasma membrane with HDAC1 stability itself regulated upstream by the VCPKMT-VCP proteasomal degradation axis. Pharmacological inhibition of each isoform disrupts the respective circuit, converging on ferroptosis sensitization across cancer types. Flat-headed arrows denote inhibition, pointed arrows denote activation. HDACi, histone deacetylase inhibitor; GSH, glutathione; GPX4, glutathione peroxidase 4; SLC7A11, solute carrier family 7 member 11; NRF2, nuclear factor erythroid 2-related factor 2; FTH1, ferritin heavy chain 1; HMOX1, heme oxygenase 1; FSP1, ferroptosis suppressor protein 1; CoQ10, coenzyme Q10; LIP, labile iron pool; m6A, N6-methyladenosine; VCPKMT, VCP lysine methyltransferase; VCP, valosin-containing protein; NSCLC, non-small cell lung cancer; CRC, colorectal cancer. Created with BioRender.com

HDAC10 modulates ferroptosis resistance in non-small cell lung cancer through a three-node epigenetic-post-transcriptional circuit. Li et al. demonstrated that HDAC10 deacetylates histone H3 lysine 9 (H3K9ac) at the miR-223-5p gene promoter, thereby repressing miR-223-5p transcription. As miR-223-5p acts as a direct transcriptional and post-transcriptional repressor of SLC7A11, the maintenance of H3K9 acetylation by HDAC10 ultimately sustains SLC7A11 protein expression and ferroptosis resistance (Shi et al., 2024[42]). Genetic silencing of HDAC10 in A549 and H1975 cells resulted in reduced levels of GSH and SLC7A11, alongside increased intracellular iron content, ROS, and ACSL4 expression, which promote ferroptosis. This effect was reversed by re-expression of SLC7A11 or inhibition of miR-223-5p, thereby confirming the three-node mechanistic architecture (Shi et al., 2024[42]). This study presents the inaugural experimental characterization of the epigenetic-microRNA-transporter axis involving a specific HDAC isoform and its role in ferroptosis resistance in lung cancer. The findings suggest that HDAC10 may serve as a potential therapeutic target to restore ferroptosis sensitivity in NSCLC (Figure 3(Fig. 3)).

## Cancer-Type Specific Evidence

The sensitizing effects of HDAC inhibitors on ferroptosis are not universally observed across all tumors. Instead, these effects manifest in cancer-specific molecular contexts, which are determined by the particular HDAC isoforms expressed, the active ferroptosis resistance pathways in each cancer type, and the degree of iron-dependence within the cancer cell population. The available preclinical evidence specific to each cancer type is summarized in the following subsections.

### Lung cancer

Lung cancer is the most studied type of cancer in regards to the HDACi-induced ferroptosis sensitization. Zhang et al. reported that lung adenocarcinoma cells resistant to intrinsic and acquired EGFR-TKI exhibit increased sensitivity to the ferroptosis inducers erastin and RSL3. Furthermore, they found that vorinostat significantly enhances this sensitivity by downregulating the expression of SLC7A11 (xCT), leading to a reduction in intracellular GSH and a marked decrease in lipid peroxide accumulation (Zhang et al., 2021[60]). This negative correlation between EGFR-TKI resistance and ferroptosis sensitivity - the most TKI-resistant cells being the most ferroptosis-sensitive confirmed this relationship across a series of LUAD cell lines, making ferroptosis induction with HDACi a useful therapeutic application to a clinically intractable resistance phenotype (Sheng et al., 2025[40]). In non-small cell lung cancer (NSCLC), HDAC10 has been identified as the isoform responsible for maintaining SLC7A11 expression through epigenetic regulation via the miR-223-5p axis. The knockdown of HDAC10 restored sensitivity to ferroptosis by increasing intracellular levels of iron, reactive oxygen species (ROS), and ACSL4 in A549 cells (Shi et al., 2024[42]). Also, panobinostat and vorinostat could be used together with GPX4 inhibitor RSL3 to selectively kill drug-tolerant persister cells derived in response to erlotinib of NSCLC in a mitochondrial ROS-dependent pathway, but spared parental non-persister cells and offered a cancer-cell-selective combination approach to targeting minimal residual disease (Higuchi et al., 2026[15]).

### Colorectal cancer

Colorectal cancer contains the richest mechanistic information of HDAC-ferroptosis interactions of solid tumors. HDAC3-NRF2-GPX4 antiferroptotic axis regulates intrinsic ferroptosis resistance in CRC cells, and their targeted knockout using RGFP966 or pan-HDACi generates potent ferroptotic cell death with Fe2+ accumulation and lipid ROS and morphological changes of the mitochondrial membrane typical of ferroptosis (Jin et al., 2025[21]). The VCPKMT-VCP-HDAC1 axis of the control of iron storage in FTH1, in its turn, in CRC, is disrupted which allows more labile iron to be released into the cells and predisposes them to erastin (Huang et al., 2025[17]). Yang et al. reported a third independent mechanism, which is lactylation of HDAC1 at lysine 412, which stabilizes the expression of FSP1 through m6A-mediated mRNA stabilization; HDACi treatment restores this lactylation and decreases FSP1 levels, making cells in CRC cells sensitive to ferroptotic agents such as RSL3, erastin, and FINO2 (Yang et al., 2025[57]). The identification of multiple HDAC-regulated ferroptosis resistance pathways in colorectal cancer (CRC), specifically HDAC3, HDAC1, and HDAC1 lactylation, underscores the significant role of epigenetic mechanisms in ferroptosis resistance within this malignancy. Furthermore, it highlights the considerable potential of HDAC inhibitor (HDACi)-based sensitization strategies.

### Gastric cancer

In gastric cancer, ferroptosis sensitization through HDACi has been directly reported by Jenke et al. that demonstrated vorinostat and entinostat induce lipid peroxidation and ferroptotic cell death in various GC cell lines including cisplatin-resistant variants (Jenke et al., 2024[18]). The main mechanism that was observed was SLC7A11 suppression that caused GSH depletion, and proteomic analysis showed a widespread reorganization of the lipid metabolism and iron-handling protein networks in line with multi-target ferroptosis sensitization (Jenke et al., 2024[18]). The pronounced dependence of gastric cancer (GC) on SLC7A11-mediated cystine import for cisplatin resistance underscores its vulnerability to therapeutic strategies involving HDAC inhibitors, which aim to restore ferroptosis sensitivity in models of chemoresistant disease (Zhang et al., 2025[63]).

### Hematological malignancies

In hematological cancers, the HDAC inhibitors regulate ferroptosis sensitivity by mechanisms that supplement the already known direct cytotoxic and differentiation-inducing actions. The HDAC inhibitor treatment in AML increases the labile iron pool by reducing iron storage capacity and the blockage of iron export, which produce conditions that boost ferroptosis along with GSH-depleting agents (Bian et al., 2025[2]). HDAC inhibitors also repress transcription of pro-survival genes in leukemic cells and make them sensitive to ferroptosis inducers by lowering the GPX4 expression threshold necessary to execute ferroptosis (Hosseini et al., 2024[16]). The immediate implementation of HDACi-ferroptosis combination strategies in clinical trials for acute myeloid leukemia (AML) and other leukemia subtypes is now feasible, owing to the clinical availability of pan-HDACi with well-established hematological safety profiles.

### Glioma

Glioma presents distinct pharmacological challenges, including the necessity for blood-brain barrier penetration and the prevention of HDACi-induced ferroptosis resistance. However, it also offers unique therapeutic opportunities, as malignant glioma cells exhibit a high dependency on GPX4 and SLC7A11 to evade ferroptosis. In contrast, normal neurons, which are generally resistant to HDACi-induced ferroptosis, are also typically resistant to HDACi-induced ferroptosis sensitization (Ma et al., 2024[31]).The HDAC inhibitor vorinostat has been reported to induce ferroptosis in GBM cells by downregulating SLC7A11 and depleting GSH simultaneously, without triggering neurotoxic ferroptosis in non-malignant neuronal populations - an effect that is selective to the expression of HDAC isoforms between tumor and normal brain cells, and the capacity of GSH reserves to do so (Ma et al., 2024[31]). Dual-mechanism HDAC-ferroptosis hybrid molecules that are the first-in-class to achieve cancer cell selectivity over normal lung fibroblasts (WI-38) and retinal pigment epithelial cells (RPE) in NCI-H522 NSCLC and HCT-116 CRC models have also been demonstrated, and the platform can be used to develop a pharmacological template applicable to glioma-targeted drug development (Karaj et al., 2022[22]) (Table 1(Tab. 1); References in Table 1: Bian et al., 2025[2]; Higuchi et al., 2026[15]; Hosseini et al., 2024[16]; Huang et al., 2025[17]; Jenke et al., 2024[18]; Jin et al., 2025[21]; Karaj et al., 2022[22]; Ma et al., 2024[31]; Sheng et al., 2025[40]; Shi et al., 2024[42]; Yang et al., 2025[57]; Zhang et al., 2021[60]; Zhang et al., 2025[63]).

## Combination Strategies and Hybrid Molecules

The mechanistic convergence of HDAC inhibition and ferroptosis induction has led to the development of three pharmacological strategies that extend beyond single-agent ferroptosis sensitization. These strategies include the design of first-in-class dual-mechanism HDAC-ferroptosis hybrid molecules, the combination of approved HDAC inhibitors with GPX4 inhibitors or system Xc- antagonists, and the integration of HDAC inhibitor-mediated ferroptosis with immune checkpoint blockade.

### First-in-class dual-mechanism HDAC-ferroptosis hybrid molecules

The pioneering bifunctional hybrid molecules, as described by Karaj et al., are characterized by their ability to simultaneously induce ferroptosis and inhibit HDAC proteins within a single chemical structure. This is achieved through the conjugation of the pharmacophores of vorinostat (SAHA), a pan-HDAC inhibitor, and CETZOLE, a ferroptosis-inducing agent (Karaj et al., 2022[22]). The lead hybrid HY-1 is shown to have potent cancer cell cytotoxicity with GI50 values as low as 20 nM in NCI-H522 NSCLC and HCT-116 CRC cells, and it is selective against cancer cells over normal WI-38 lung fibroblasts and RPE retinal epithelial cells - where it is hypothesized to be selective due to cancer cells highly expressing thioredoxin to counter HDACi-induced ROS (Karaj et al., 2022[22]). The fact that HY-1 caused lipid peroxide accumulation measured as C11-BODIPY fluorescence, increased transferrin receptor protein, which is one of the established markers of ferroptosis, and caused caspase-3 cleavage as a result of HDAC inhibition was confirmed through mechanistic validation making it possible to identify dual-pathway cell death as the mechanism of its action (Karaj et al., 2022[22]). Hybrid molecules have the pharmacological benefit of having homogeneous spatiotemporal distribution of their two active pharmacophores which inhibits the pharmacokinetic disparity that can negate two-drug combination schedules in vivo and provides simultaneous target absorption at the tumor site.

### HDACi combined with GPX4 inhibitors

The combination of approved HDAC inhibitors with GPX4 inhibitors or system Xc- antagonists represents the most clinically viable strategy for inducing synergistic ferroptosis in cancer phenotypes that exhibit resistance to therapy. Higuchi et al. showed that, at sub-cytotoxic levels as low as those required to achieve standalone cell death by itself, pre-treatment of drug-tolerant lung, melanoma and breast cancer persister cells with either panobinostat or vorinostat primed the persister cells to subsequent ferroptotic killing by GPX4 inhibitor RSL3, and parental sensitive cells were largely spared (Higuchi et al., 2026[15]). The time-based strategy involving HDACi pretreatment followed by GPX4 inhibition demonstrates maximal selectivity for inducing ferroptosis in persister cells while minimizing systemic toxicity. This approach directly addresses a significant challenge in the clinical application of ferroptosis-based therapies. Additionally, in the context of colorectal cancer (CRC), another proposed therapeutic strategy involves the combination of HDAC3 inhibitors with erastin or RSL3 to induce ferroptotic cell death in treatment-resistant cells. These cells rely on the HDAC3-NRF2-GPX4 axis for survival. Notably, a synergistic ferroptotic effect is observed in the combination of RGFP966 and RSL3 across various CRC cell lines (Jin et al., 2025[21]).

### HDACi combined with immune checkpoint inhibitors

HDAC inhibitors independently restructure the tumor immune microenvironment by enhancing MHC-I antigen presentation, facilitating CD8+ T-cell infiltration, reducing PD-L1 nuclear translocation, and re-polarizing tumor-associated macrophages to the pro-inflammatory M1 macrophage phenotype. These modifications collectively potentiate the efficacy of anti-PD-1/PD-L1 checkpoint blockade (Zhang et al., 2025[61]). These immune effects are further amplified by ferroptotic cell death which releases damage-associated molecular patterns (DAMPs) and oxidized lipid species, which activate the innate immune response and increase tumor immunogenicity, which leads to a self-reinforcing loop of ferroptotic death-induced immunogenicity and immune-mediated tumor clearance (Tian et al., 2025[47]). Clinical trials with HDACi prior to anti-PD-1 antibodies have shown better survival and disease control rates on refractory tumors such as colon cancer, melanoma, and pancreatic cancer and, in addition, HDACi pre-treatment enhanced the invasion of the tumor microenvironment by CD8+ cytotoxic T-cells and decreased the proportion of regulatory T-cells (Tian et al., 2025[47]). The integration of HDACi-ferroptosis-immunotherapy triplet combinations represents a mechanistically coherent and translationally promising therapeutic strategy, warranting prioritization in forthcoming clinical trials (Yu et al., 2024[58]).

## Challenges and Future Directions

Despite robust preclinical evidence supporting the concept, the integration of ferroptosis sensitization via HDAC inhibitors into clinical practice encounters a range of interconnected scientific, pharmacological, and translational challenges that must be systematically addressed.

### Isoform selectivity and off-target toxicity

The primary challenge in translating current research into clinical practice is the lack of isoform selectivity among approved histone deacetylase (HDAC) inhibitors. Pan-HDAC inhibitors, such as vorinostat and panobinostat, inhibit nearly all zinc-dependent HDACs simultaneously. This broad inhibition results in dose-limiting myelotoxicities, including thrombocytopenia, neutropenia, cardiotoxicity, and severe fatigue, which restrict their application in solid tumors but permit their use in hematological contexts (Shi et al., 2024[42]). As HDAC isoforms regulate different ferroptosis regulation axes - HDAC3 through the NRF2-GPX4 hierarchy (Liang et al., 2023[28]), HDAC10 through the miR-223-5p/SLC7A11 pathway (Jin et al., 2025[21]), and HDAC1 by FTH1 and FSP1 (Huang et al., 2025[17]) - isoform-selective (rather than global) inhibitors are a more specific pharmacological approach. RGFP966 and emerging class I-selective benzamides are HDAC3-selective inhibitors with enhanced tolerability but do not lose ferroptosis-sensitizing potency in preclinical models of CRC and NSCLC (Zhang et al., 2025[64]). The next-generation HDAC inhibitors are being developed as proteolysis-targeting chimeras (PROTACs) that can selectively degrade a single HDAC isoform, not all of them, which should be considered a promising direction toward better selectivity with minimal off-target toxicity (Banerjee et al., 2026[1]; Hawash, 2025[12]; Smalley et al., 2020[45]).

### Context-dependent roles and cancer-specific validation

The different tissue types have context-dependent opposing functions of various individual HDAC isoforms, which is a key issue. Even though HDAC3 supports ferroptosis resistance in CRC (Jin et al., 2025[21]), HDAC3 knockdown has been reported to suppress ferroptosis in non-cancer inflammatory disease models (Shi et al., 2025[41]), which demonstrates that the ferroptosis-sensitizing activity of HDACi is not universal and needs to be validated in cancers before clinical applications. Another example of factors that can regulate ferroptosis sensitivity is tumor microenvironment factors; hypoxia, nutrient deprivation, stromal interactions, should be included in the development of combination strategies (Gao et al., 2025[9]; Szwed et al., 2025[46]). Recent discoveries on heterogeneity in ferroptosis susceptibility within intratumors, based on single-cell and spatial transcriptomic, further complicate predictions of therapeutic response using bulk tumor analysis (Kim et al., 2026[23]; Lee et al., 2025[26]).

### Biomarker development and patient stratification

The fact that predictive biomarkers of HDACi-ferroptosis sensitivity would not be validated is also a major limitation of clinical translation. Each of the following factors including SLC7A11, GPX4, ACSL4, HDAC3, and ferroportin-based on ferroptosis sensitivity has been shown to be mechanistically correlated with prognostic outcome in retrospective cancer variables: The level of SLC7A11 expression, the amount of GPX4 protein, the level of ACSL4 expression, the activity of HDAC3, and the status of ferroportin (He et al., 2023[13]; Lee et al., 2025[26]; Ou et al., 2024). They are urgently required to be validated as companion biomarkers to stratify the patients when putting HDACi-ferroptosis combination trials into practice (Zhang et al., 2024[62]; Zhang et al., 2025[64]).

### Drug delivery and in vivo translation

In vitro cell line models have been the most common preclinical ferroptosis sensitization models in which drug levels and the availability of iron in the cell lines vary significantly compared to the levels and availability in vivo tumors (Wen et al., 2019[49]; Yang et al., 2020[55]). One of the new ways to address this translational gap is nanoparticle-based delivery platforms that co-deliver HDAC inhibitors and ferroptosis inducers - allowing dual simultaneous release of both agents into the tumor (Li et al., 2024[27]; Xiang et al., 2024[51]). Nanoparticles of iron oxides capable of expanding intracellular labile iron pool and delivering HDAC inhibitors have enhanced ferroptosis levels in xenograft models compared to their individual agents and liposomal formulations of vorinostat with GPX4 inhibitors have demonstrated improved pharmacokinetic stability and accumulation in the tumor of NSCLC mouse models (Xu et al., 2025[53]; Zhou et al., 2024[65]). Regularity concerns of nanomaterial-based combinations also require strict characterization of important quality features and biodistribution profiles prior to investigational new drug use (Szwed et al., 2025[46]).

### Clinical trial design

Lastly, rational combination of HDACi-ferroptosis sensitization combinations in clinical trials will necessitate a solution to optimal timing, namely, whether HDACi pretreatment prior to ferroptosis inducer delivery maximizes persister cell selectivity (Higuchi et al., 2026[15]) or whether one should select HDACi dose as well as patient stratification based on ferroptosis biomarker status and incorporation of pharmacodynamic endpoints, e.g., circulating 4-HNE-protein adducts or plasma malondialdehy (Zhou et al., 2024[68]). As a priority next stage in clinical translation of this epigenetic anticancer strategy, first-in-human studies of dual-mechanism HDAC-ferroptosis hybrid molecules (Tian et al., 2025[47]) and isoform-selective HDAC3 inhibitors in ferroptosis-sensitization indications should be conducted.

## Conclusion

The accumulating evidence presented in this review substantiates that HDAC inhibitors are mechanistically diverse epigenetic agents capable of dismantling the ferroptosis-resistant phenotype of cancer cells through four convergent and non-redundant molecular pathways: the silencing of SLC7A11 transcription leading to subsequent GSH depletion, the destabilization of intracellular iron homeostasis via the downregulation of ferroportin and expansion of the labile iron pool, the enhancement of mitochondrial ROS production which amplifies lipid peroxidation, and the silencing of the HDAC3-NRF2-GPX4 antiferroptotic hierarchy.

It is crucial to note that these processes do not represent class generalization; rather, they are mediated by cancer type-specific regulatory loops involving distinct HDAC isoforms (HDAC1, HDAC3, HDAC10) in colorectal, lung, gastric, and hematological cancers. This isoform selectivity has the potential to transform HDAC inhibition from a nonspecific epigenetic intervention into a rationally targetable pharmacological strategy.

The biological foundation of therapeutic relapse, characterized by the selective vulnerability of drug-tolerant persister cells to HDACi-primed ferroptosis, represents a highly promising translational opportunity within the field. The integration of established epigenetic pharmacology, advanced molecular ferroptosis biology, and combination immunotherapy has positioned HDAC inhibitor-induced ferroptosis sensitization as an innovative therapeutic model to address drug resistance in cancer. Rigorous clinical research employing isoform-selective agents, validated biomarkers, and rational combination regimens will be essential to realize this potential in patient treatment.

## Declaration

### Conflict of interest

The authors declare that they have no competing interests.

### Acknowledgments

The authors extend their appreciation to Prince Sattam bin Abdulaziz University for funding this research work through the project number (PSAU/2025/03/39106).

### Funding

This study was supported by funding from Prince Sattam bin Abdulaziz University (project number: PSAU/2025/03/39106).

### Author Contributions

Mubarak A. Alamri: Conceptualization, data curation, writing the original draft. Muhammad Afzal: Visualization, writing the original draft. Surya Nath Pandey: Data curation, writing the original draft. Mohamed Ahmed Akela: Formal analysis, writing - review and editing. A Rekha: Conceptualization, writing the original draft. All authors have approved the final version of this manuscript.

### Availability of data and materials

Not applicable.

### Consent for publication

Not applicable.

### Ethics approval and consent to participate

Not applicable.

### Artificial Intelligence (AI) - assisted technology

The authors confirm that no artificial intelligence (AI) technologies, such as large language models (LLMs), chatbots, or AI-based image generators, were utilized in preparing this manuscript. The scientific data were generated without the use of AI tools, and the images, references, results interpretation, and modifications to the scientific conclusions were all conducted manually. All figures in this article were created using BioRender (BioRender.com) through a licensed academic subscription. The authors bear full responsibility for all scientific content, textual writing, data interpretation, and conclusions.

## Figures and Tables

**Table 1:**
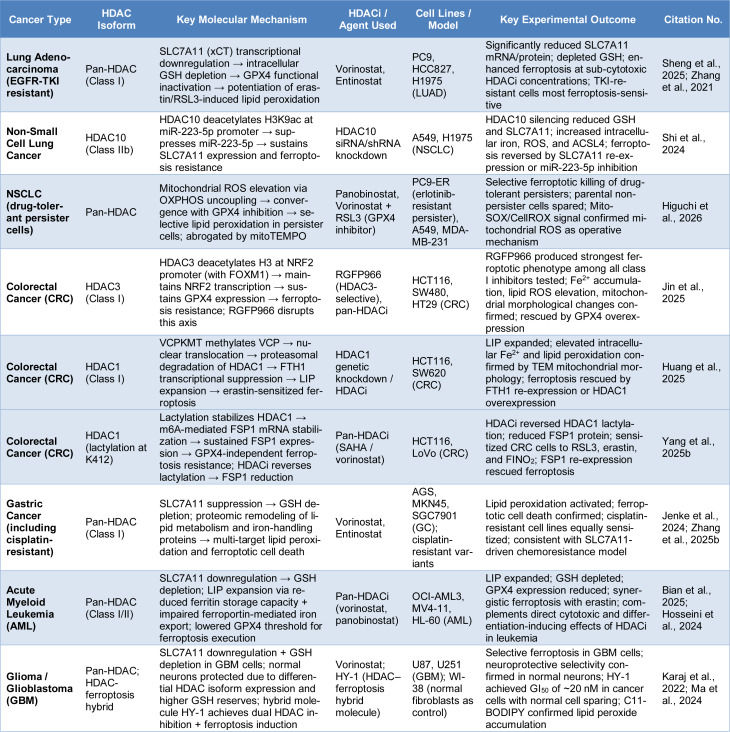
Cancer-type specific evidence of HDAC inhibitor-mediated ferroptosis sensitization

**Figure 1:**
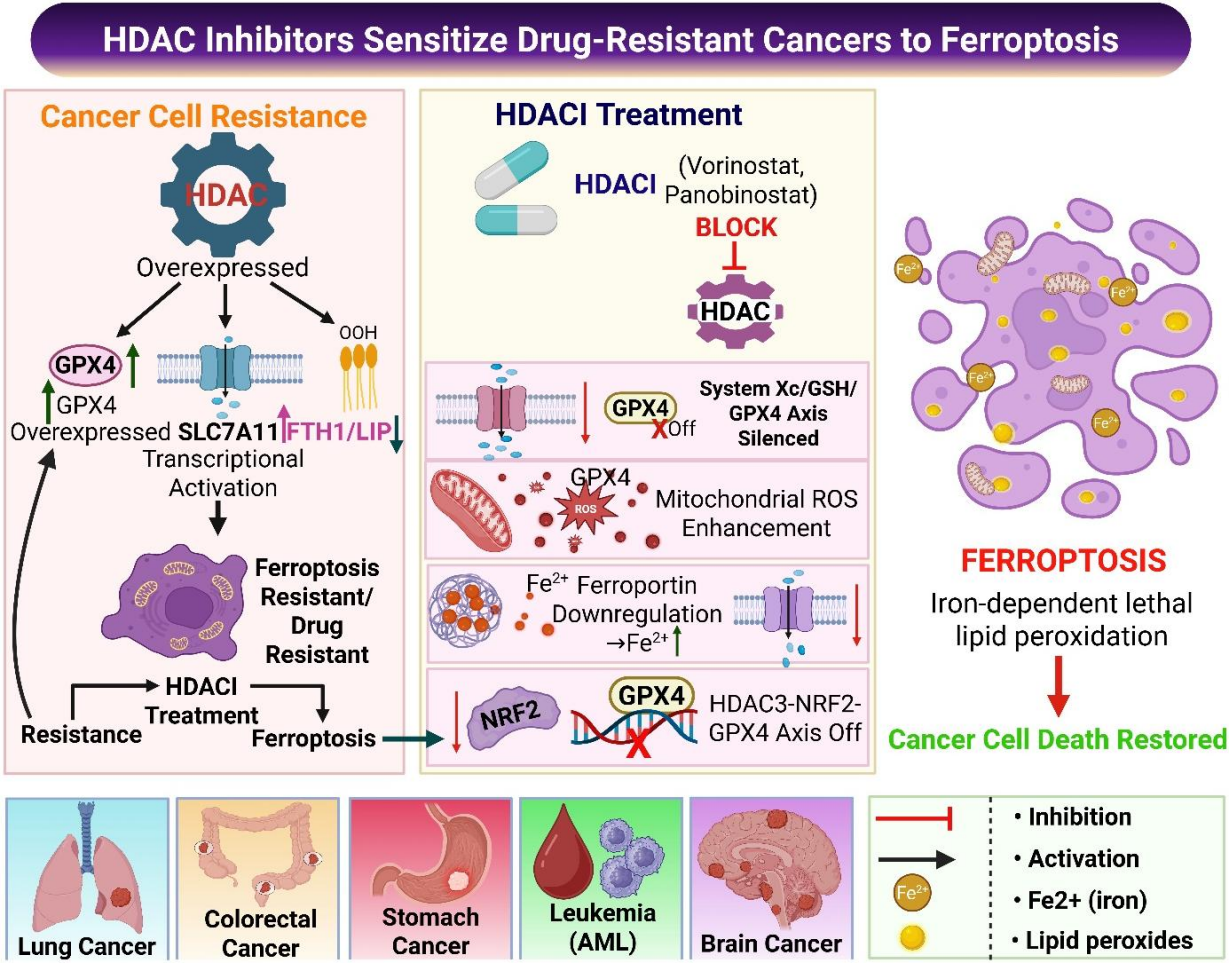
Graphical abstract
